# Analysis of Top-Down Cracking in Asphalt Pavements Based on Energy Principles

**DOI:** 10.3390/ma18071586

**Published:** 2025-04-01

**Authors:** Xiong Tao, Aoyang Zhan, Xuan Huang, Tao Bai

**Affiliations:** 1 Hubei Provincial Expressway Industrial Development Co., Ltd., Wuhan 430000, China; tx900702@163.com; 2 School of Civil Engineering and Architecture, Wuhan Institute of Technology, Wuhan 430205, China; baigs08@wit.edu.cn; 3 Hubei Communications Investment Xiangyang Expressway Operations Managements Co., Ltd., Xiangyang 441199, China; hx995249@163.com

**Keywords:** asphalt pavement, top-down cracking (TDC), fracture mechanics, dissipated strain energy, numerical simulation, aging, ultraviolet (UV), anti-aging agent

## Abstract

This study develops a fracture energy-based finite element model to assess top-down cracking (TDC) in asphalt pavements, integrating aging, healing, and interlayer bonding effects through viscoelastic fracture mechanics. Computational analyses of typical Chinese materials and structures reveal that SMA-13 surface layers improve TDC resistance by 19.8% compared to conventional AC-25 mixtures in 17 cm thick pavements. Thicker asphalt layers (20 cm) extend crack initiation life by 16.2% under standard axle loads. UV radiation reduces TDC life by 1.55–2.60%, concentrating 82% of cracks in wheel-path zones. Anti-aging agents restore 47% of fracture energy loss, maintaining stable energy dissipation ratios (EDR > 0.75) beyond 50 months. Poor bonding consumes 19.1% of TDC life, with crack density in wheel paths 3.2× higher than in non-wheel areas. Critical thresholds are identified: longitudinal wheel-path zones require 12% higher fracture energy to prevent crack initiation compared to transverse zones. The model demonstrates that combining ≥18 cm asphalt layers, polymer-modified surfaces (PG76-22), and chemical stabilizers (e.g., 1.5% Sasobit) reduces aging-induced TDC risks by 34–41%. These findings provide mechanics-based guidelines for designing durable pavements in freeze-thaw regions.

## 1. Introduction

Cracking is one of the common distresses occurring in asphalt pavements, with causes including temperature fluctuations, aging, traffic loading, environmental/climatic issues, etc. [1]. Load-related fatigue cracking can be further divided into bottom-up cracking and Top-Down cracking (TDC). In traditional pavement structural designs, it is often desired to cost-effectively optimize the pavement structure whilst maximizing and sustaining its longevity and endurance against load-related distresses, such as cracking, during its design life. For cost-competitiveness purposes, the pavement thickness tends to be thinner, making bottom-up and reflection crack prevention a crucial issue. As the durability and longevity requirements for pavements continue to be enhanced, there will be a convergence to an appropriate increase in the asphalt layer thickness. Correspondingly, there will also be an inherent increase in the proportion of TDC occurrence [2]. Currently, China’s asphalt pavement design standards [3] are predominantly focused on preventing bottom-up reflection cracks, with the governing design criteria being the bending horizontal-tensile strains at the bottom of the hot-mix asphalt (HMA) layer. To these authors’ best knowledge, at the time of writing this paper, no standardized design criteria yet exist in China for mitigating and/or limiting TDC in asphalt pavements.

In the 1980s, TDC was successively reported in South Africa, France, and the Netherlands [4]. This phenomenon was later observed and reported in Japan, the United Kingdom, and the United States in the early 21st century [4]. Researchers speculate that factors such as asphalt mixture design volumetrics, traffic loading, thermal stresses, aging, etc., are related to this phenomenon. Among the existing mature design methods, the American mechanistic-empirical (M-E PDG) method calculates fatigue damage using Miner’s law and then quantitatively determines the TDC life from the fatigue damage [5,6,7]. However, this model does not distinguish the two stages of crack initiation and crack propagation distinctively. In addition to this method, there are other approaches, such as the viscoelastic continuous damage model [8,9,10,11] and the micromechanical model [12,13,14,15] for conducting TDC analysis. The former model utilizes pseudo-stiffness as the calculation index and considers the healing effect of viscoelastic damage and micro-damage of the asphalt mixture. This model can be employed to determine the spatial distribution of damage within a fixed timeframe, evaluate the concentration of fatigue damage (i.e., above and/or below the asphalt layer) for top-down fatigue cracking, and observe the evolution of damage over time. However, it does not consider the aging condition of the asphalt mixture and the actual traffic-loading spectrum. Additionally, it has not been locally calibrated, and its current form is relatively complex for routine practical use.

On the other hand, the latter model is inclined to strictly describe the initiation stage of top-down cracks at a microscopic scale. This includes considering the contact characteristics between aggregates, the non-uniformity of materials, and the influence of voids in the asphalt mixture. However, it is important to note that the actual validity of the model cannot be determined on the macroscopic scale. Based on the principles of viscoelastic fracture mechanics, the University of Florida [14,16,17,18] developed the Florida top-down fracture model (HMA-FM). This model posits that the transverse tensile stress generated by radial tires on pavement surfaces is chiefly responsible for causing cracking. This issue is closely associated with the observed longitudinal cracking of the outer non-wheel path band measuring 0.5 m—although it does not correspond with the longitudinal cracking of the pavement surface beneath the wheel track path. To this end, the Florida Team has introduced the concept of energy ratio, in combination with the dissipative creep strain energy limit, to design the pavement thickness and predict pavement life for top-down cracking in Florida [14,16,17,18]. This concept considers the influence of pavement structural characteristics and the impact of creep properties of the asphalt mixtures on the top-down cracking phenomenon. However, simplifying the model whilst ensuring prediction accuracy remains one of the key challenges.

The effects of healing and degradation of the viscoelastic material properties due to aging over the life of the pavement have not been fully considered, thus limiting the HMA-FM’s ability to assess the TDC resistance of asphalt pavements. Therefore, Yared [19] proposed a new set of material sub-models based on the asphalt mixture morphology. These sub-models consider the changes in key asphalt mixture properties caused by the effects of aging and healing on damage accumulation. Additionally, they predict the cracking time of TDC induced by load using comprehensive asphalt mixture characteristics and material properties. To further enhance the predictive accuracy of the model, Yared and Birgisson et al. [20] incorporated the axle load spectrum into the analytical framework based on fracture mechanics concepts. They also established the impact of various traffic characteristic parameters, such as vehicle category distribution, traffic seasonal variation, traffic growth rate, and lateral wheel drift, on the predictive performance of fatigue cracking. To account for the viscoelastic plasticity of the asphalt mixture, a crack initiation criterion based on energy was proposed for asphalt mixtures, drawing from Griffith’s crack initiation criterion for elastic materials [21]. To address certain limitations of the proposed TDC model, Gu et al. [22,23] conducted a TDC study based on different generational locations whilst also considering the impact of aging and healing. So far, research on TDC has progressed through the stages of application and development of linear elastic fracture mechanics, fatigue fracture mechanics, and viscoelastic fracture mechanics. Currently, the focus of most research regarding asphalt pavement cracking is mostly on the application of fatigue fracture mechanics theory and related methods.

In the HMA energy-based model and fracture mechanics framework, the macroscopic crack development at any time during crack initiation is described using some upper and lower energy thresholds [24]. These threshold limits are represented by the Dissipated Creep Strain Energy (DCSE) and Fracture Energy (FE), respectively [25]. Once the energy threshold is surpassed, non-healing macroscopic cracks will form and propagate throughout the asphalt mixture. On the other hand, the rheological properties of the asphalt mixture cause stress redistribution in the pavement system, thereby accumulating viscoelastic residual stress under continuous repeated loading [26]. The viscoelastic properties of the asphalt mixture should thus be considered for the asphalt pavement cracking model to be more accurately representative and effective [27]. The study of HMA fracture mechanics indicates that the limit of DCSE in asphalt mixtures effectively defines the threshold for fracture damage [28,29].

In this study, an energy model developed based on the principles of material viscoelasticity and fracture mechanics was utilized to characterize and analytically quantify TDC on a hypothetical municipal road at different locations, including approximating the crack initiation life. The main objective of the study was to model and numerically characterize the cracking mechanism of TDC in typical pavement structures and determine the HMA material and structural composition that can better resist top-down fatigue cracking. The research findings are envisioned to serve as a valuable reference datum for mitigating TDC and designing crack-resistant asphalt surfaces for typical pavement structures in Urumqi, China.

## 2. Top-Down Crack Initiation Modeling

**Figure 1:**
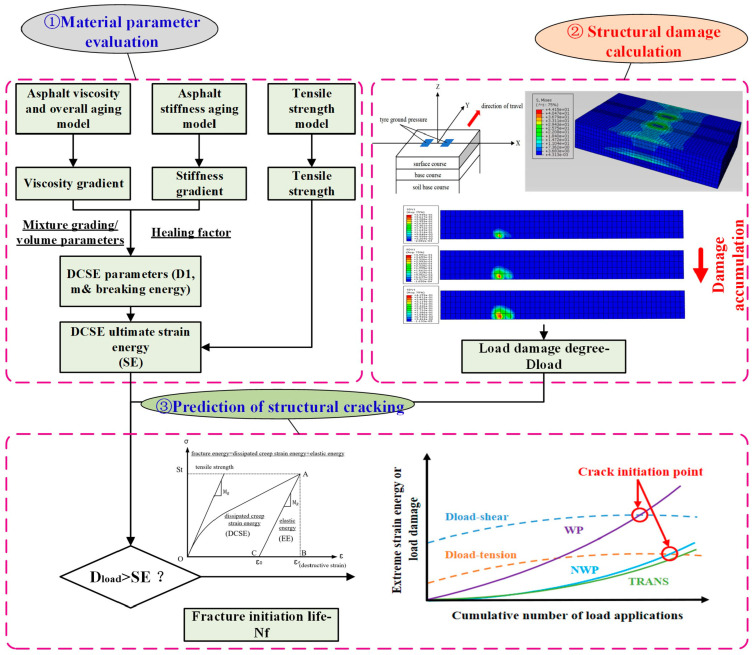
Study flow plan for energy-based TDC model assessment.

The rheological properties of the asphalt binder and the volumetric characteristics of the asphalt mixture were utilized to estimate the creep compliance parameters of the asphalt mixture. Crack analysis was conducted based on the different locations of the TDC manifestation. When the damage to the asphalt pavement caused by loading exceeds the yield strain energy of the asphalt mixture, crack initiation will commence on the pavement surface, and thus, it becomes possible to predict the TDC initiation life at different locations within the pavement structure. Among these crack types/classes, TDC is divided into longitudinal wheel path cracking (shear stress-related), longitudinal non-wheel path cracking (transverse tensile stress-related), and transverse cracking (longitudinal tensile stress-related) [30]. The research concept and study flow chart for the fracture energy-based top-down crack initiation model, as evaluated herein, is diagrammatically illustrated in Figure 1.

## 3. Pavement Structural Model Characterization

The three-dimensional (3-D) finite element (FE) model of the asphalt pavement was established using the ANSYS software (13.0) [31] to quantitatively analyze the mechanical responses of the pavement structure. Firstly, the load damage caused by different stress responses was calculated by inputting the key stress-related inputs into the fatigue damage model and combining them with the creep parameters of the surfacing asphalt mixture. Secondly, the material parameters representing the material properties were input into the TDC model to obtain the DCSE of the asphalt mixture under different fracture modes, which was used to reflect the ultimate strain energy of the asphalt mixture with respect to resisting cracking. Lastly, the crack initiation life of the asphalt pavement was predicted according to the crack criterion that cracks occur when the pavement load-related damage reaches (and/or exceeds) the ultimate strain energy of the material, namely the asphalt mixture.

### 3.1. Finite Element (FE) and Fatigue Damage Analysis

#### 3.1.1. FE Force-Loading Configuration

**Figure 2:**
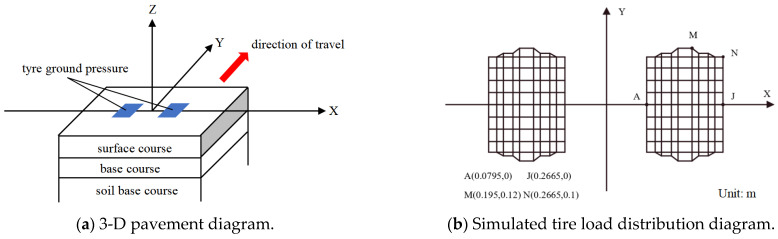
The 3-D pavement structure model and surface loading configuration.

The analyzed hypothetical pavement model comprised three layers: asphalt layer, base layer, and soil base layer. Figure 2 depicts the schematic diagram of the 3-D FE model and the nodal diagram for non-uniform tire loading input. To input the viscoelastic parameters of the asphalt mixture, the surface layer utilized the viscoelastic unit Visco89 from ANSYS [31]. This unit adopts the form of the generalized Maxwell model [32] that allows for a more accurate representation of the material properties. Simultaneously, the surface asphalt layer was segmented into sub-layers to account for the temperature variations/gradient more effectively. The 3-D solid unit Solid 95 with intermediate nodes was employed in the base, while Plane 82 with 8 nodes was utilized in the plane unit. The grid was divided into hexahedrons and locally subdivided into tetrahedrons.

**Figure 3:**
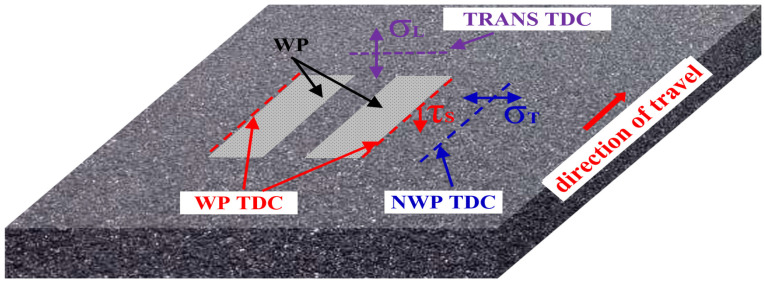
The 3-D pictorial view of the stress response points on the pavement surface.

The pavement structure was modeled as a square-shaped slab with dimensions of 5 m × 5 m for both length and width, whilst the soil foundation beneath it had a thickness of 6m. To simulate the loading and stress distribution on an actual pavement structure, the measured tire-pavement contact pressure, including the vertical, lateral, and horizontal directional stress, was used. For the specific model configuration, boundary conditions, and interlayer contact conditions, references should be made to these authors’ previous publications, including those of Mao et al. [33] and Bai et al. [34,35]. Figure 3 illustrates the calculation and response locational positions for the longitudinal wheel path (WP), longitudinal non-wheel path (NWP), and transverse track zone (TRANS) in the driving direction.

#### 3.1.2. Structural Fatigue Damage Analysis

From a fracture energy perspective, tensile and shear stresses have a damaging effect on asphalt pavements. When the fracture damage accumulates above the material’s yield limit, cracks will appear on the pavement surface. The TDC model utilized in this paper proposes an energy damage model for the tensile shear failure under each dynamic load cycle as follows [22]:

(1) $$D_{t/c}=\int_{0}^{0.1}\sigma_{0}\sin\left(10\pi t\right)\varepsilon_{p\max}\sin\left(10\pi t\right)dt$$

(2) $$D_{s/c}=2\left(1+\nu \right)\int_{0}^{0.1}\tau_{s}\sin\left(10\pi t\right)\varepsilon_{p\max}\sin\left(10\pi t\right)dt$$

(3) $$\varepsilon_{p\max}=m\times D_{1}\times 1000^{\left(m-1\right)}$$

where, $D_{t/c}$ is the damage caused by the tensile stresses of each load cycle, $\sigma_{0}$ is the tensile stress at the critical position, $D_{s/c}$ is the damage caused by the shear stresses of each load cycle, $\tau_{s}$ is the shear stress at the tire edge, ν = 0.35 is the Poisson ratio of the asphaltic material, and *t* is the loading time varying from 0 to 0.1 s. The parameter $\varepsilon_{p\max}$ is the maximum creep strain rate of the asphalt mixture, whilst *m* and *D*_1_ are the creep compliance parameters. The total load damage caused by the tensile-shear stress is expressed by Equations (4) and (5) below [22]:

(4) $$D_{\mathrm{load},t}=D_{t/c}\times N_{c}$$

(5) $$D_{\mathrm{load},s}=D_{s/c}\times N_{c}$$

where, $D_{\mathrm{load},t}$ and $D_{\mathrm{load},s}$ are the load damage caused by the total tensile stress and the load damage caused by the total shear stress, respectively, and $N_{c}$ is the number of dynamic load cycles.

### 3.2. Top-Down Cracking Modeling

**Figure 4:**
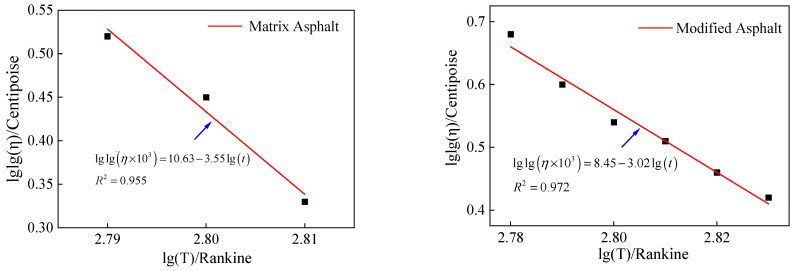
Urumqi’s asphalt viscosity-temperature curve.

In this study, the stiffness gradient of the asphalt binder was determined based on the viscosity and aging model of the asphalt binder. The dynamic modulus and tensile strength were then calculated based on the volumetric parameters of the asphalt mixture. At the same time, the creep compliance parameters can be simultaneously derived from fitting the coefficients of the dynamic modulus master curves, which are the relevant DCSE parameters of the asphalt mixture. The viscosity-temperature curve of Urumqi’s asphalt is shown in Figure 4.

#### 3.2.1. Asphalt Viscosity-Aging Model

(1) binder aging viscosity

The change in the binder viscosity is directly related to the dynamic modulus and creep characteristics of both the binder and asphalt mixture. The binder viscosity can be estimated from the A-VTS model using Equation (6) [36]:

(6) $$\log\log\left(\eta_{0}\right)=A+VTS\times \log\left(T_{R}\right)$$

where, $\eta_{0}$ is the initial viscosity of the binder in Centipoise (cP), $T_{R}$ is the Rankine temperature whilst A and VTS are the regression constants.

For determining the binder viscosity after a certain period of aging, the aging model can be utilized to make an estimation using Equation (7) [37]:

(7) $$\log\left(\log\eta_{aged}\right)=\frac{\log\left(\log\eta_{t=0}\right)+At}{1+Bt}$$

where,

(8) $$A=-0.004166+1.41213\times C+\log\left(MAAT\times \frac{9}{5}+32\right)\times C+\log\left(\eta_{t=0}\right)\times D$$

(9) B=0.197725+0.068384×logC

(10) $$C=10\left[274.4946-193.831\times \log T_{R}+33.9366\times {\left(\log T_{R}\right)}^{2}\right]$$

(11) $$D=-14.5521+10.47662\times \log T_{R}-1.88161\log{T_{R}}^{2}$$

And $\eta_{aged}$ is the viscosity of the binder after aging (cP), MAAT is the average annual temperature (°C), and t is the aging time (months).

(2) binder aging stiffness

An aging model for the binder stiffness was utilized to calculate the stiffness of the binder under different aging conditions. Within this model, the change in the binder stiffness is mathematically considered to be linearly correlated with the change in the logarithm of the binder viscosity, as expressed by Equation (12) [38]:

(12) $$S\left(t\right)=S_{0}\times \frac{\log\eta_{t}}{\log\eta_{0}}$$

where, S(t) is the asphalt-binder stiffness (stiffness modulus) at aging time *t*, $S_{0}$ is the initial asphalt-binder stiffness prior to aging, $\eta_{t}$ is the asphalt-binder viscosity at aging *t*, and $\eta_{0}$ is the initial asphalt-binder viscosity prior to aging.

Considering that asphalt mixtures have the capability to self-heal during the pavement’s service life, the healing ability of the asphalt mixture is thus inherently related to the asphalt-binder stiffness and fracture energy. In this study, the healing factor was determined as expressed in Equation (13) [39,40,41]:

(13) $$h_{y}\left(t\right)=1-C_{h}\times {\left[S_{n}\left(t\right)\right]}^{\frac{FE_{i}}{1.67}}$$

where: $h_{y}\left(t\right)$ is the healing factor at aging time *t*, $S_{n}\left(t\right)$ is the aging coefficient of the asphalt-binder stiffness at aging time *t*, $FE_{i}$ is the initial fracture energy of the asphalt-binder, and $C_{h}$ is the adjustment factor with a numerical value of 1.0.

(3) Ultraviolet Aging Effects

**Figure 5:**
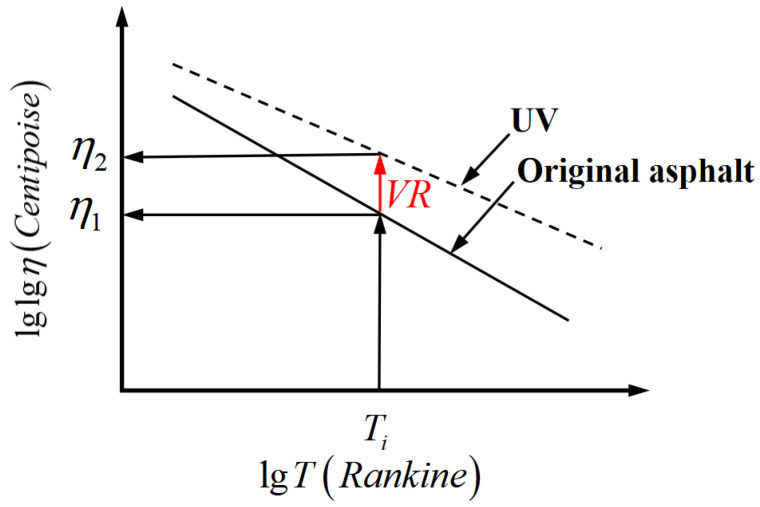
Asphalt-binder viscosity diagram.

As illustrated in Figure 5, the logarithmic viscosity ratio under different aging conditions was used to calculate and quantitatively characterize the decay in the asphalt-binder viscosity at any given temperature using Equation (14) [26]:

(14) $$\omega =\mathrm{lg}VisRatio=\frac{\mathrm{lg}\eta_{1}}{\mathrm{lg}\eta_{2}}$$

From the literature [42,43], the average viscosity ratios of the asphalt binders after ultraviolet aging at different temperatures are summarized in Table 1.

In this study, the logarithmic viscosity ratio ω was introduced to improve the adhesive viscosity and the overall aging model, as shown in Equation (15):

(15) $$\eta_{aged}=10\wedge \left[10\wedge \left(\frac{\log\left(\log\eta_{t=0}\right)+At}{1+Bt}\right)\right]\omega$$

#### 3.2.2. Stiffness and Strength of the Asphalt Mixture

(1) Dynamic modulus characterization

The dynamic modulus $\left|E^{*}\right|$ of the asphalt mixture can be determined through dynamic compression experiments conducted at various temperatures, resulting in the generation of a master curve at different loading frequencies. In the absence of experimental and laboratory test data, $\left|E^{*}\right|$ can be estimated using the following time-temperature dependent S-shaped logarithmic model expressed in Equation (16) [44]:

(16) $$\log\left|E^{*}\right|=\delta +\frac{\alpha }{1+e^{\left(\beta +\gamma \log t_{r}\right)}}$$

where $\left|E^{*}\right|$ represents the compression dynamic modulus (psi), $t_{r}$ is the reduction time (s) at the reference temperature, whilst δ and α are the shape fitting parameters that depend on the asphalt mixture volumetrics such as aggregate type/gradation, asphalt-binder type/content, density (air voids), etc. For a given set of data, δ represents the minimum value of $\left|E^{*}\right|$ whilst δ+α represents the maximum value of $\left|E^{*}\right|$. Additionally, β and λ are the shape-fitting parameters that describe the shape of the S-type logarithmic function and partly depend on the viscosity of the asphalt-binder. In this study, the modified shape-fitting parameters δ, α, β, and λ were used as follows [45]:

(17) $$\delta =2.718879+0.079524\times \rho_{200}-0.007294\times {\left(\rho_{200}\right)}^{2}+0.002085\times \rho_{4}-0.01293\times V_{a}+0.08541\frac{V_{be}}{V_{be}+V_{a}}$$

(18) $$\alpha =3.559267-0.005451\times \rho_{4}+0.020711\times \rho_{3/8}-0.000351\times {\left(\rho_{3/8}\right)}^{2}+0.00532\times \rho_{3/4}$$

(19) β=−0.513574−0.355353×log(η)

(20) γ=0.37217

where $V_{a}$ is the air void ratio; $V_{be}$ is the effective asphalt-binder content (%); $\rho_{3/4}\left(\rho_{3/8},\rho_{4}\right)$ are the aggregate-mass percentage (%) passing the 19 mm, 9.5 mm and 4.75 mm sieve seizes, respectively; $\rho_{200}$ is the aggregate-mass percentage (%) passing through the 0.075 mm screen size; and η is the viscosity of the asphalt-binder.

(2) Tensile strength characterization

Based on the correlation between the tensile strength and tensile stiffness of the asphalt mixture, the following nonlinear regression model was employed to obtain the tensile strength of the asphalt mixture [1]:

(21) $$S_{t}=\sum_{n=0}^{5}a_{n}{\left(\log S_{f}\right)}^{n}$$

(22) $$S_{f}=\lambda_{r}\times \left|E^{*}\right|$$

where, $S_{t}$ is the tensile strength (MPa); $S_{f}$ is the tensile stiffness (MPa); and $\left|E^{*}\right|$ is the dynamic modulus of the asphalt mixture (MPa). The value of the reduction coefficient $\lambda_{r}$ used was 0.4 whilst the constant $a_{n}$ had values as follows: $a_{0}=284.01$, $a_{1}=-330.02$, $a_{2}=151.02$, $a_{3}=-34.03$, $a_{4}=3.7786$, $a_{5}=-0.1652$ [16].

(3) Creep compliance parametric characterization

Creep compliance is a fundamental property that describes the relationship between the time-varying strain and applied stresses in viscoelastic materials. The creep compliance curve can be utilized to represent the time-varying characteristics of the asphalt-binder and asphalt mixture, respectively, as well as to evaluate the damage accumulation rate in the asphalt mixture. The determination and characterization of the creep compliance of the asphalt mixture is crucial for evaluating the cracking performance of asphalt pavements. As reported in some of the literature [46], the creep compliance parameters, *m* and *D*_1_, are also highly correlated with the dissipated creep energy of the asphalt mixture.

**Figure 6:**
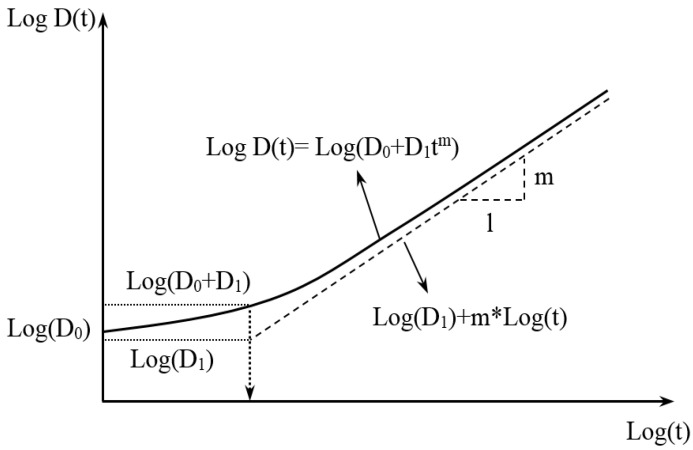
Creep compliance function and associated parameters [18].

The creep compliance function, D(t), and its corresponding parameters are depicted in Figure 6 below. In the figure, $D_{0}$ represents the instantaneous elastic response, while $D_{1}$ it denotes the initial segment of the creep compliance curve, and *m* signifies the slope of the longer period within the same curve.

Two methods exist in the literature that commonly determine the creep compliance parameters. The first method is based on the reciprocal relation of $D\left(t\right)=1/\left|E^{*}\right|$, which is obtained by transforming the dynamic modulus master curve. The second method involves direct estimation based on the rheological properties of asphalt-binder and the viscoelastic characteristics of the asphalt mixture using the following model relationships [45]:

(23) $$\log\left(D_{0}\right)=-\delta -\alpha -\log\lambda_{r}$$

(24) $$\log\left(D_{0}+D_{1}\right)=-\delta -\frac{\alpha }{1+e^{\beta }}-\log\lambda_{r}$$

In the above equations, the reduction coefficient $\lambda_{r}$ used in this study was 0.4, and parameters δ, α and β, γ can be obtained from the asphalt mixture volumetrics and the asphalt-binder viscosity using Equations (17)–(20). The slope of $\log t-\log\left|E^{*}\right|$ curve when *t* = 1800 s is typically taken as the *m*_0_ value after reducing the |E*| master-curve function to Equation (25) [45]:

(25) $$m_{0}=\alpha \gamma \times \frac{e^{\left(\beta +3\gamma \right)}}{{\left[1+e^{\left(\beta +3\gamma \right)}\right]}^{2}}$$

After considering the additional viscosity term, namely the change in the asphalt-binder viscosity due to aging effects, the prediction equation for the m value transforms to Equation (26), where κ is a constant with a numerical value of 0.408 [22].

(26) $$m=m_{0}+\frac{\kappa }{\log\log\eta }$$

#### 3.2.3. Strain Energy Quantification

**Figure 7:**
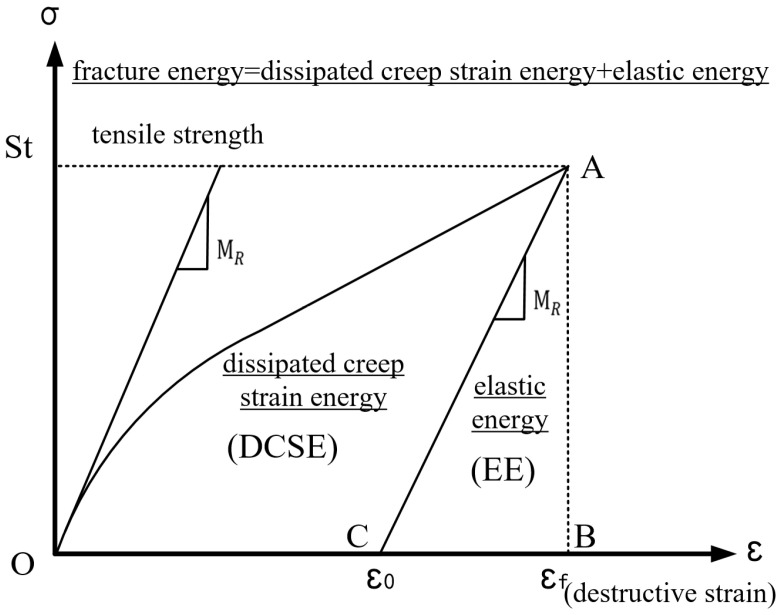
Graphical representation of DCSE [9].

The development of fatigue cracks in this study was quantified using the DCSE limit concepts, which was calculated as the difference between the breaking/fracture energy (FE) and the elastic energy (EE)—see Figure 7. The fracture energy represents the total area under the stress-strain response curve [47,48], whilst the elastic energy corresponds to the triangular area enclosed by the elastic stress-strain response curve. The quantitative threshold for DCSE signifies the amount of energy that an asphalt mixture can endure before fracturing. For any given asphalt mixture, DCSE generally increases with an increase in the loading time. Theoretically, a higher creep compliance value (which is the inverse of the stress relaxation rate) in magnitude results in a greater rate of accumulation for the DCSE and vice versa. Consequently, asphalt mixtures with quantitatively higher DCSE thresholds will mathematically demonstrate superior resistance to cracking compared to those with lower DCSE thresholds under similar environmental and loading conditions. Therefore, DCSE can potentially serve as an indicative measure for evaluating top-down cracking performance in asphalt mixtures.

In this study, the concept of DCSE was utilized to quantitatively characterize the strain energy of the asphalt mixture in response to induced cracking. It is important to consider that the fracture energy, tensile strength, resilience modulus, and stiffness of the asphalt mixture all undergo changes over time during the aging process of the asphalt binder. As a result, the DCSE threshold $DCSE_{\lim}$ becomes a function of time, *t*, as the independent variable, and is mathematically expressed as follows [45]:

(27) $$DCSE_{\lim-T}=FE_{t}-\frac{{S_{t}}^{2}}{\left(2\times MR_{t}\right)}$$

(28) $$FE_{t}=FE_{i}-\left(FE_{i}-FE_{\min}\right){\left[S_{n}\left(t\right)\right]}^{k}$$

(29) $$S_{n}\left(t\right)=\frac{S\left(t\right)-S_{0}}{S_{\max}-S_{0}}$$

where $DCSE_{\lim-T}$ represents the ultimate strain energy of the asphalt mixture during tensile fracture; $FE_{t}$ denotes the fracture energy per unit length of the asphalt mixture at aging time, *t*; and $FE_{i}$ signifies the initial fracture energy per unit length. $FE_{\min}=0.2\;kJ/m^{3}$ stands for the residual fracture energy per unit length of aging and $S_{t}$ indicates the tensile strength of the asphalt mixture at aging time, *t*. $MR_{t}$ represents the elastic modulus of the asphalt mixture at aging time, *t*. $S_{n}\left(t\right)$ is defined as the aging coefficient of the asphalt-binder at aging time, *t*; S(t) is the stiffness of the asphalt mixture that changes with aging time, *t*; $S_{0}$ represents the initial stiffness of the asphalt mixture, $S_{\max}$ denotes the maximum stiffness of the asphalt mixture after a long time of aging, *t*; and k=4.0 is the aging constant [22].

Based on the crack initiation criterion of fracture mechanics, the ultimate strain energy for tensile fracture differs from that of shear fracture (i.e., shear stress-induced fracture). The conversion formula for the ultimate strain energy of the asphalt mixture’s shear fracture based on tensile fracture is as follows:

(30) $$DCSE_{\lim-S}=C_{f}\times DCSE_{\lim-T}$$

where $DCSE_{\lim-S}$ is the ultimate strain energy of the asphalt mixture’s shear stress-induced cracking, and $C_{f}=2.0$ is the adjustment factor, which correlates the ultimate strain energy of tensile fracture to that of shear fracture. The absolute threshold of DCSE can be determined using the above equations, which inherently signifies the energy characteristics of an asphalt mixture in relation to its cracking resistance potential.

### 3.3. Energy Ratio Characterization

The generation and propagation of cracks in asphalt mixtures are partly a function of the energy threshold and vary greatly from one type of asphalt mixture to another [49,50]. However, the energy threshold is not necessarily correlated with the creep compliance characteristics of asphalt mixtures, and thus, it is not possible to accurately predict the cracking performance solely based on the performance of a single asphalt mixture. Considering the significant influence of the pavement structural and creep compliance characteristics on the pavement cracking performance, Roque et al. [17] introduced the energy ratio (ER) concepts into the fracture mechanics model for asphalt mixtures. ER is a dimensionless parameter defined as the DCSE threshold of the asphalt mixture divided by the required dissipative variable strain energy minimum and is mathematically calculated as expressed in Equation (31) [17].

(31) $$ER=DCSE_{f}/DCSE_{\min}$$

With,

(32) $$DCSE_{\min}=m^{2.98}\times D_{1}/f\left(S_{t},\sigma_{\max}\right)$$

(33) $$f\left(S_{t},\sigma_{\max}\right)=150059.1\times {\sigma_{\max}}^{-3.10}\times \left(6.36-S_{t}\right)+2.46\times 10^{-8}$$

where $DCSE_{f}$ represents the threshold value of the DCSE, $S_{t}$ denotes the tensile strength (MPa), $\sigma_{\max}$ is the maximum tensile stress (MPa), $f\left(S_{t},\sigma_{\max}\right)$ is a function of the tensile strength and tensile stress in the asphalt pavement, and $DCSE_{\min}$ indicates the minimum DCSE required when cracking appears.

Based on the fundamental fracture mechanics theory and as reported in some of the literature [51], some correlations exist between fracture energy and material properties, such as the tensile strength, modulus, etc., with respect to fracture damage. In their publication, Monteiro et al. [52], in fact, proposed using material strength, elastic modulus, and fracture energy for designing asphalt mixtures. Therefore, in lieu of the above equations and considering the fracture energy/damage versus material properties correlations, these authors proposed and utilized the following model formulation for $DCSE_{f}$ estimation:

(34) $$DCSE_{f}=FE-S_{t}^{2}/\left(2\times MR\right)$$

where FE is the fracture energy of the asphalt mixture ($kJ/m^{3}$); $S_{f}$ is the tensile stiffness (MPa), and MR is the elastic modulus of the asphalt mixture (MPa).

## 4. FE Modeling, Simulations, and Numerical Results

### 4.1. Pavement Structural Configuration

**Figure 8:**
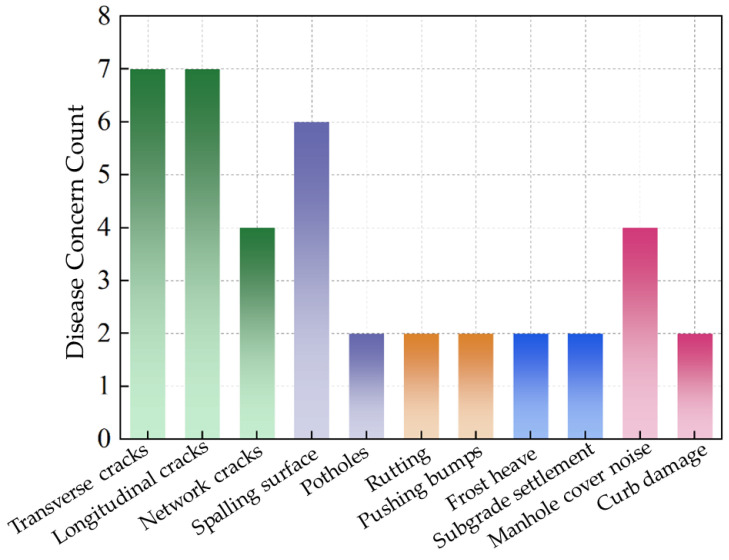
Disease Concern Count.

Based on the 2021 field performance monitoring and surface distress surveys conducted in Urumqi (China), it was found that cracking accounted for approximately 65% to 75% of the total pavement distress measured/recorded. See Figure 8.

From the field surveys, the most observed cracking type was top-down cracking. Figure 8 exemplifies a typical pavement structure for the main local municipal road consisting of an asphalt macadam SMA-16 (surfacing layer), asphalt concrete AC-20, and asphalt stabilized macadam ATB-25 as the total 20 cm asphalt mixture thickness.

**Figure 9:**
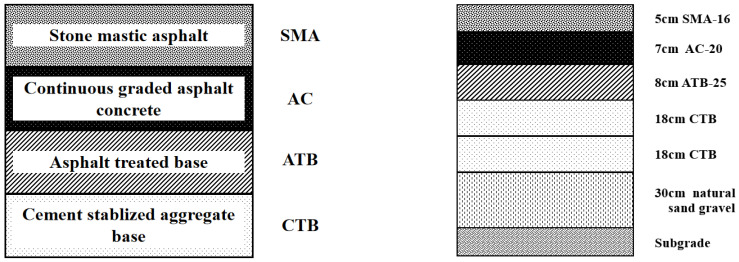
Typical municipal road-pavement structure.

**Figure 10:**
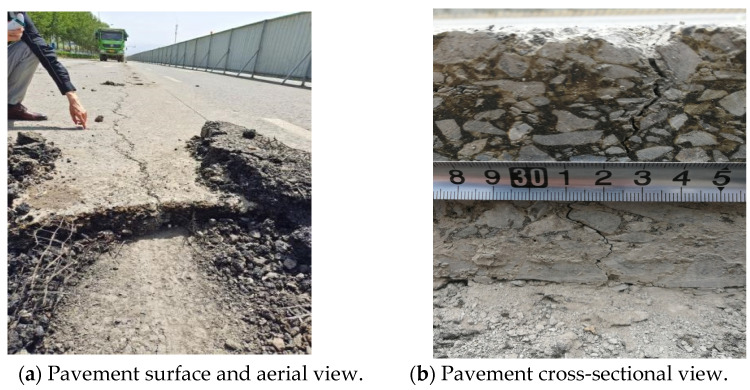
Photographic pictures of typical top-down cracking on Urumqi roads.

As illustrated in Figure 9, the base layer comprises a two-layer cement-stabilized sand gravel semi-rigid structural layer, totaling 36 cm thick. The sub-base comprises 30 cm of natural sand gravel resting on an in-situ compacted subgrade soil material. Figure 10 exemplifies typical top-down cracking distresses (geographic coordinates: 87.632° E, 43.817° N) observed in Urumqi, China.

In Figure 10, the longitudinal cracks are concentrated in a continuous band along the wheel path (WP), with wider cracks at the top and narrower cracks at the bottom (see Figure 10b). From pavement cross-sectional saw-cuttings and forensic evaluations, these cracks were visually observed to extend through the middle and upper surface layers, i.e., they extended to various depths within these layers. The prevalence of these cracks along the WP suggests that traffic wheel loading is one of the primary causes.

Field investigations by these authors found aggregate segregation with the AC-10/13 mixture comprising mostly of the fines, whilst the SMA-16 had more of the medium-sized grain aggregates. During core sampling, it was observed that the underlying ATB layer was loosely compacted and could not be fully extracted [53]. Therefore, the rehabilitation and reconstruction of the test section prioritized the replacement of SMA-16 with SMA-13, ATB-25 with AC-25 for the lower layer, and utilizing modified asphalt binder for the upper surfacing layer. Additionally, an anti-aging agent was added to the upper surfacing layer, whilst an anti-spalling agent was added to the lower layer. To offset the cost increment resulting from these material changes/replacements, an attempt was simultaneously made to reduce and optimize the thickness of the asphalt layers without compromising the pavement’s structural integrity and overall performance. Additionally, to investigate the effectiveness of the reconstructed pavement structure in resisting TDC further, five different pavement transitional structures were added to the study matrix for comparison purposes. The pavement structures that were modeled and numerically evaluated are presented in Table 2. The base asphalt binder used was 90# Karamay asphalt, the modified asphalt binder was SBS grades I-B, and all the SMA mixtures were modified with lignin fiber.

**Figure 11:**
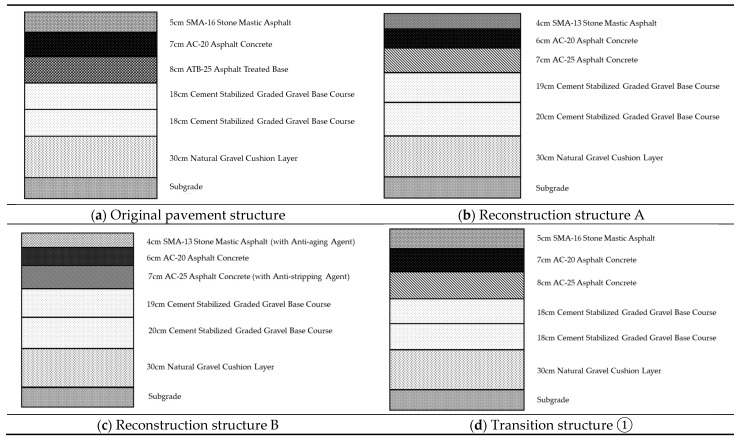

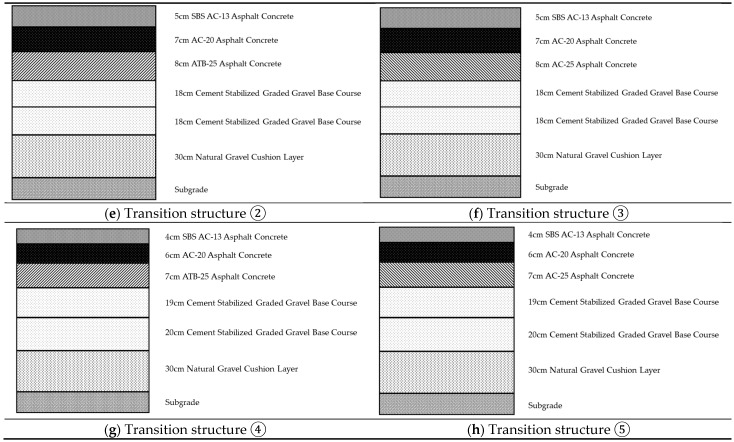
Schematic diagram of the pavement structure.

Figure 11 shows the schematic diagram of different pavement structures.

The fracture energy, resilience modulus, and dynamic modulus of the asphalt mixture, as well as its creep compliance parameters, *m* and *D*_1_, were obtained through formula deductions using Equations (16)–(30). The material input parameters used in the TDC prediction model are listed in Table 3 below.

### 4.2. Pavement Layer Thickness Combinations

**Figure 12:**
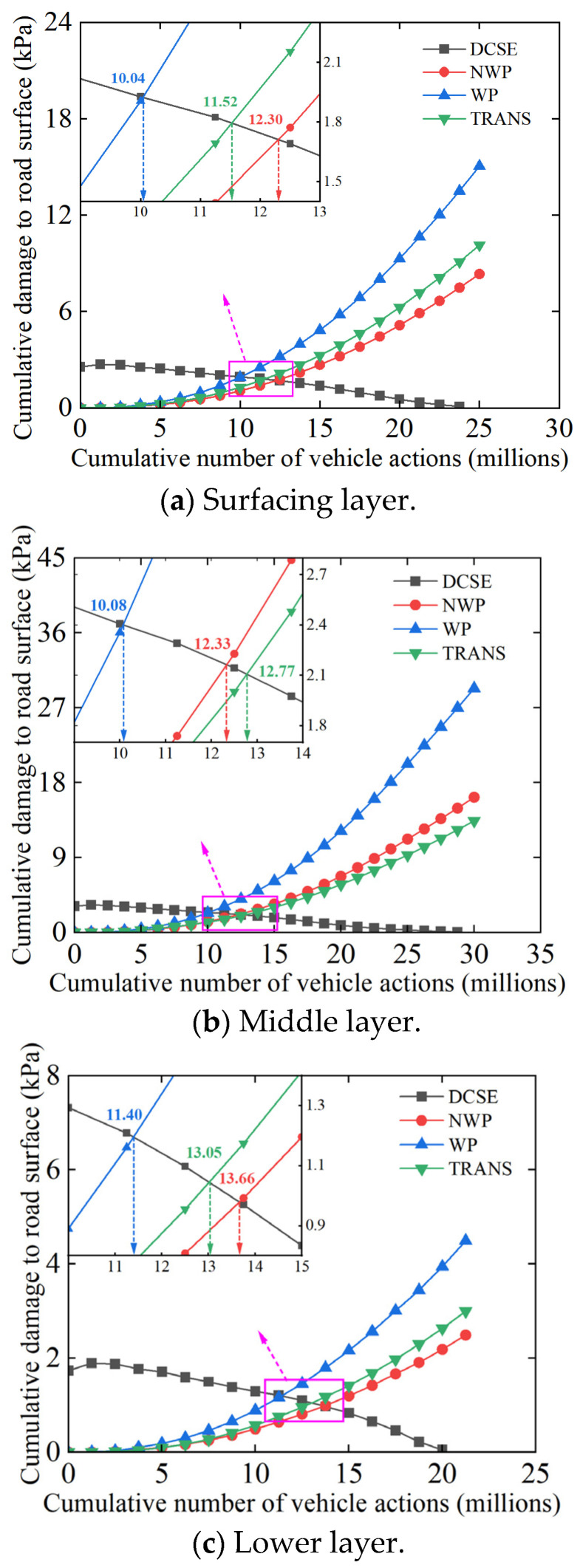
TDC in the original pavement structure.

According to the meteorological data of Urumqi (China), the annual average temperature is 7.88 °C. For the practicality of this study, 10 °C was used in the asphalt binder stiffness aging model to simulate the aging environment of the asphalt binder. When UV aging is not considered, the prediction of TDC at the key horizontal locations of each layer of the original road-pavement section is shown in Figure 12.

From Figure 12, the cracking time of the surfacing (upper) layer occurs relatively earlier than that in the middle and lower layers, respectively, which theoretically infers that the original pavement section’s cracks could be mostly top-down. This is consistent with the on-site field observations, with the cracks always occurring in the wheel path (WP) first. As theoretically expected, the TDC generation in the non-WP (NWP) zone and transverse (TRANS) position occurred much later after that in the WP.

**Figure 13:**
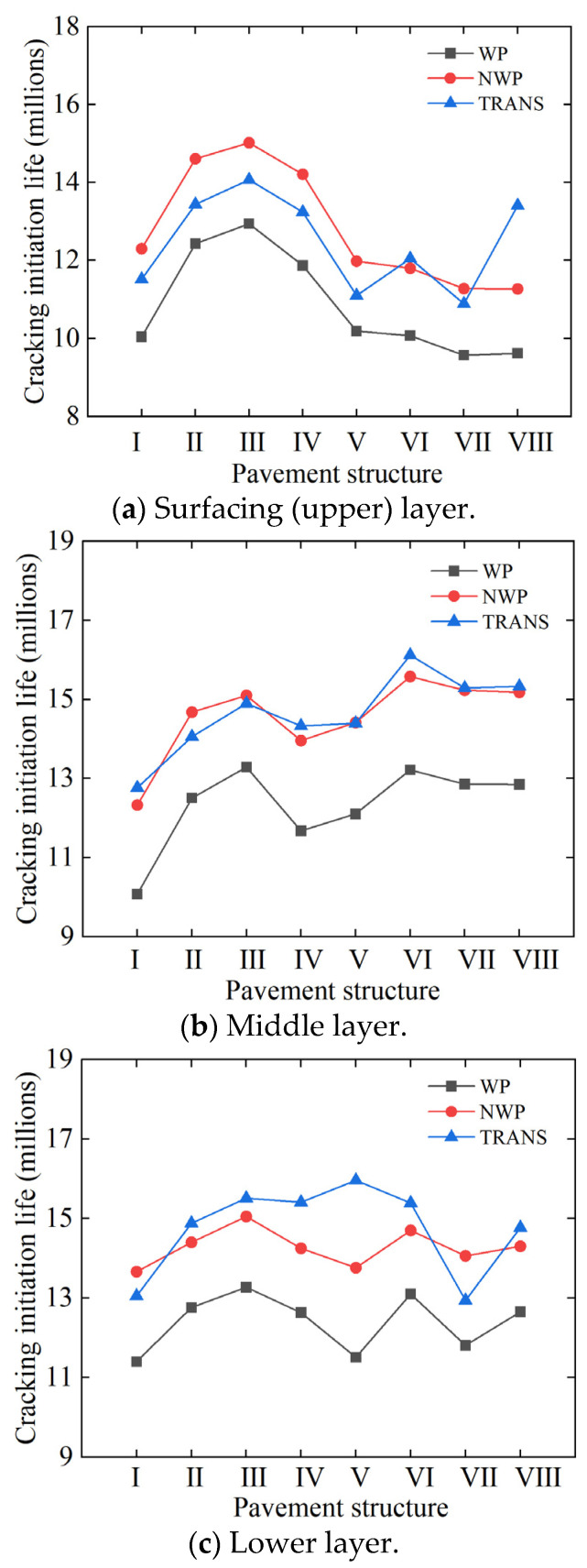
Crack initiation response trends.

The response trends of the cumulative number of load repetitions at the key crack initiation locations (namely WP, NWP, and TRANS) for different pavement structures and layers are shown in Figure 13. In particular, the figure shows that the crack initiation life in the WP and NWP changed significantly with different pavement layer thicknesses and material combinations but basically maintained a consistent response trend.

In the pavement structures with the 5/7/8 cm thickness combinations (Table 3), the TDC cracking life at the critical locations in the surfacing (upper) layer increased after replacing the ATB-25 lower layer material with AC-25, i.e., pavement structure I versus IV. The crack initiation life increment in the WP, NWP, and TRANS were 18.2%, 15.5%, and 14.9%, respectively.

After replacing the SMA-16 top surfacing (upper) layer with SAC-13 (i.e., pavement structure I versus V, VI, VII, VIII), the surfacing (upper) layer’s WP cracking life was approximately the same as before the replacement or decreased slightly, regardless of the combination of the material and structural thickness of the middle and lower layers. This analytically shows that switching to SAC-13 does not improve the cracking life of the surfacing (upper) layers. The probable reason could be that in the structural fatigue damage analysis, the loading input (namely stress) has an important influence on the accumulation rate of damage in the pavement structure. As the input stress increases, the cumulative load-related damage caused by the increasing stress also increases. According to Table 3, SAC-13 has a relatively higher modulus at 20 °C compared to SAM-16, and, thus, the pavement structure with the relatively stiffer SAC-13 as the top surfacing (upper) layer sustains higher critical stresses—but obviously lower compressive strains. In the TDC model, the asphalt mixture has a higher tensile strength gradient as the aging time changes. When considering the HMA strain energy threshold from the perspective of fracture energy, the asphalt mixture (HMA) with higher modulus has a larger stiffness aging coefficient, $S_{n}\left(t\right)$, at different aging times. This also corresponds to Figure 13a. Compared with Structure I, keeping the layer thickness and materials combination unchanged but only replacing the SMA-16 top surfacing (upper) layer with SAC-13 resulted in a declining response trend for the crack initiation life—even though the fracture energy increased. Compared to traditional AC-type asphalt mixtures, SMA-type asphalt mixtures generally have superior mix-design volumetrics with higher asphalt binder contents, typically exceeding 5.9%. Therefore, it is not theoretically surprising that switching from SMA-16 to SAC-13 led to a decline in the cracking resistance of the pavement top surfacing (upper) layer.

In the pavement structures with the 4/6/7 cm thickness combinations (Table 3), after replacing SMA-16 with the less-stiff SMA-13 (i.e., a little bit more flexible) in the top surfacing (upper) layer and ATB-25 with AC-25 in the lower layer, the TDC life of the top surfacing (upper) layer at the key response positions (i.e., WP, NPW, and TRANS) increased significantly—averaging about 19.8%. After adding an anti-aging agent and anti-peeling agent, respectively, the TDC life at the key response locations of the top surfacing (upper) layer increased even further by about 3.9%. Although the overall thickness of the surfacing (upper) layer decreased by 3 cm and correspondingly reduced the cracking life of the pavement to some extent, the use of the superior binder-rich SMA-13 asphalt mixture significantly improved the cracking resistance of the pavement surface layer. In addition to being rich in the asphalt binder content, this is partly because the SMA asphalt mixture utilized in this study comprised of a skeleton compact stone-on-stone contact structure, characterized by a clavicle frame embedded with some coarse-graded gravel aggregates, resulting in a high overall structural strength, excellent stability, and high cracking resistance potential.

In general, the reconstructed Structures II and III benefited from the superior performance advantages of the SMA-type asphalt mixture, namely SMA-13. In comparison to SMA-16 and SAC-13, which were also used as the surfacing (upper) layer materials in the other pavement structures (see Table 3), SMA-13 exhibited more flexibility with a relatively smaller modulus at 20 °C and, therefore, better resistance to cracking. Additionally, the asphalt mixture (SMA-13) demonstrated a reduced stiffness aging coefficient and tensile strength gradient over time. As a result, the cumulative rate of pavement load-related damage is more gradual. Based on Equations (13), (27) and (28), the asphalt mixture on the top surfacing (upper) layer of the reconstructed pavement structures mathematically exhibits better healing ability compared to the original and transition pavement structures, with the fracture energy depreciation rate of the asphalt mixtures being relatively flat (i.e., slope close to zero) during the aging process. According to the TDC initiation criterion, the cumulative load-related damage curve of the reconstructed pavement structures remained nearly flat and only marginally changed. However, the HMA strain energy threshold curve shows a general increase. Therefore, the crack initiation life (the intersection points between these two curves) when the cumulative damage reaches the strain energy threshold of the asphalt mixture is prolonged, as shown in Figure 13a; with the crack initiation life response curves of the pavement Structures I, II, and III showing an upward trend.

### 4.3. UV Aging Cracking Analysis

**Figure 14:**
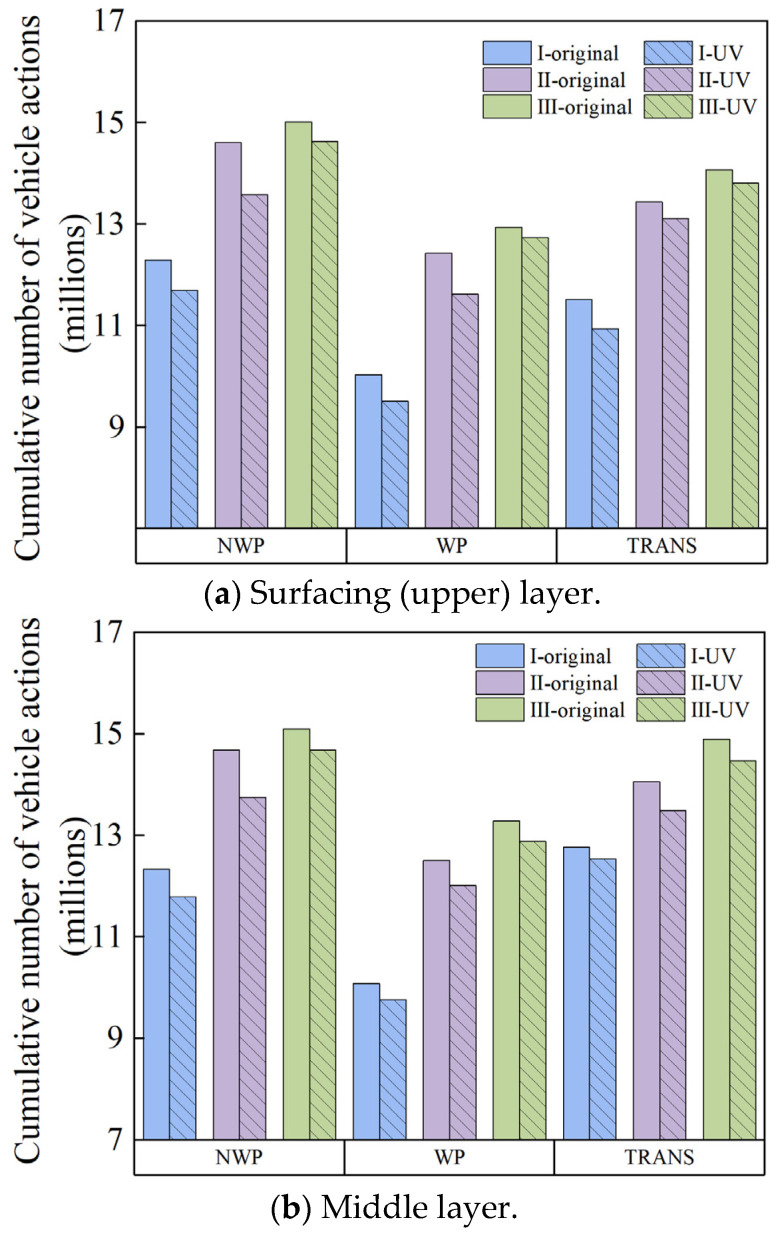

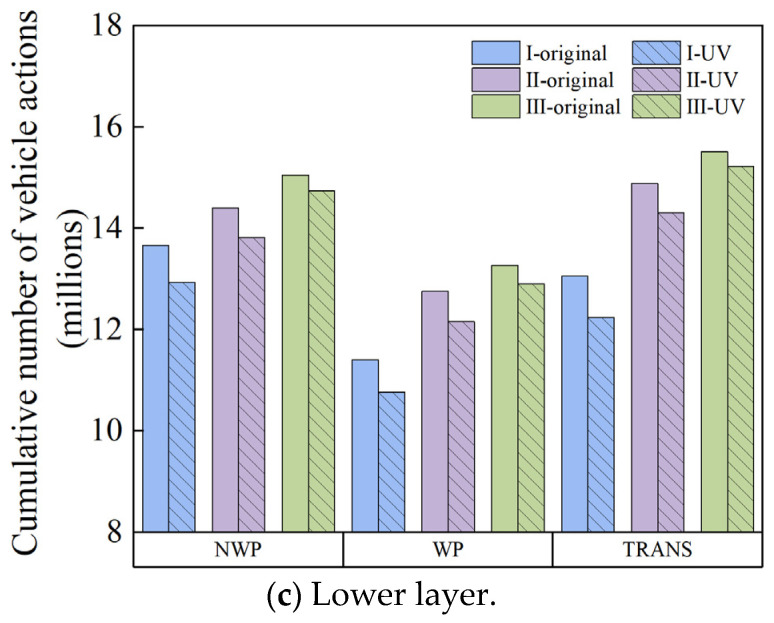
Bar chart plots of crack initiation life with and without UV aging.

When in service, asphalt pavements are subjected to prolonged exposure to light, leading to aging, age-hardening, and a decline in pavement performance [54,55,56]. According to the literature [57], the intensity of UV radiation in the Urumqi area has been gradually increasing year by year, with more than one-third of the days experiencing strong UV radiation. Considering the impact of this factor on TDC and the durability of asphalt pavements, it is imperative to consider the UV radiation environment in crack simulation models. The asphalt binder viscosity aging model considers the asphalt binder viscosity gradient under different aging times when predicting the TDC life [26]. In this modeling study, the stiffness gradient and tensile strength gradient of the asphalt mixture were inherently adjusted based on the asphalt binder viscosity gradient. Therefore, when considering the UV aging factor, the impact of UV aging on the cracking properties of the asphalt mixture is translated into its effects on the viscosity of the asphalt binder. In this study, the lifetime of cracking at each key location zone (namely WP, NWP, and TRANS) was numerically estimated and plotted in bar charts shown in Figure 14. In the figure, “original” indicates that UV radiation was not considered—whilst “UV” indicates that the effects of UV aging were considered in the modeling analysis. The notations I, II, and III refer to the pavement structures listed in Table 3, respectively.

Figure 14 illustrates that the change in cumulative pavement damage induced by loading and the variational trends without considering UV aging remained consistent. As theoretically expected, cracks first appeared in the WP zone. Under the same pavement structure, the initiation life of cracking in the different pavement layers shows a gradually increasing trend from the top surfacing (upper) layer through the middle down to the lower layer.

In the various pavement structures evaluated, the introduction of UV aging at the same key locations of the different surfacing (upper) layers resulted in a decreasing response trend in the cumulative number of sustainable load cycles, i.e., the cracking life. For example, it was observed that the cracking life of the surfacing (upper) layer of the original pavement Structure I at the key locations (namely WP, NWP, and TRANS) decreased by 4.88%, 5.18%, and 5.03%, respectively. On the other hand, the cracking life of the reconstructed pavement Structure II at the key positions decreased by 7.05%, 6.52%, and 2.46%, respectively. For the reconstructed pavement Structure III, the reductions were 2.60%, 1.55%, and 1.78%, respectively. In general, the cracking life of the original pavement structure was lower than that of the others. However, by improving the layer thickness and material combinations of the pavement structure, the cracking life tends to correspondingly improve, with the cumulative number of sustainable load repetitions increasing significantly on the reconstructed pavement structures.

As can be seen from the figure, the cracking life of the top surfacing (upper) layer of the reconstructed pavement Structure III was further increased due to the reconstructed pavement Structure II. For the top surfacing (upper) layers of the reconstructed pavement Structures II and III, the reduction in the cracking life at the critical locations before and after UV aging is 7.05%, 6.52%, and 2.46%; and 2.60%, 1.55%, and 1.78%, respectively. Evidently, these data indicate that when an anti-aging agent is included, the reconstructed pavement Structure III exhibits superior resistance to cracking in UV radiation environments than the reconstructed pavement Structure II. This is partly attributed to the enhanced flexibility of the asphalt mixture due to the addition of the anti-aging agent that inherently delays the stiffness age-hardening and possible increase in modulus caused by prolonged aging—ultimately improving the asphalt mixture’s anti-cracking properties.

The rheological properties and performance characteristics of the asphalt binder play a pivotal role in determining the quality and durability of asphalt mixtures, particularly with respect to aging and cracking. The incorporation of an anti-aging agent effectively minimizes the aging rate of the asphalt binder and ensures sustained durability, thereby significantly enhancing the anti-aging performance of the asphalt pavement and reducing its susceptibility to environmental degradation. This ultimately leads to enhancing the asphalt pavement’s overall cracking resistance potential.

### 4.4. Interlayer Bonding and Crack Analysis

Studies [21] have shown that the pavement structural stress state tends to deteriorate if interlayer debonding occurs. In this study, three interlayer contact surfaces were numerically modeled and quantitatively analyzed in each pavement structural layer. These three-layer interfaces were: (a) the bottom of the asphalt (HMA) surfacing (upper) layer and the top of the asphalt mid-surface layer; (b) the bottom of the asphalt mid-surface layer and the top of the asphalt sub-layer; and (c) the bottom of the asphalt sub-layer and the top of the base layer, respectively.

**Figure 15:**
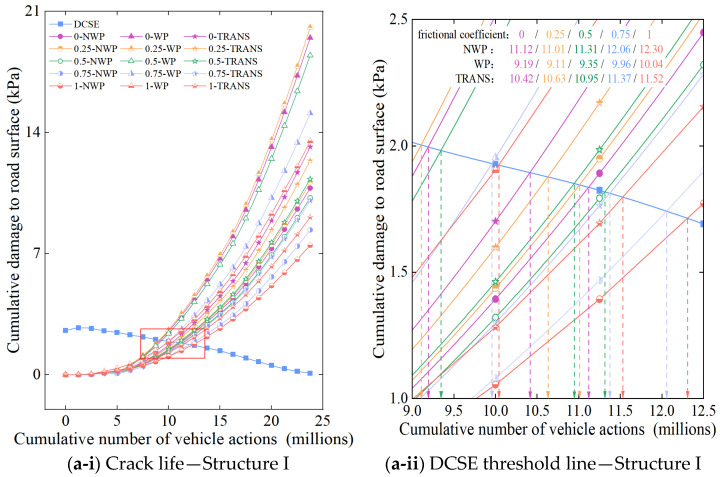

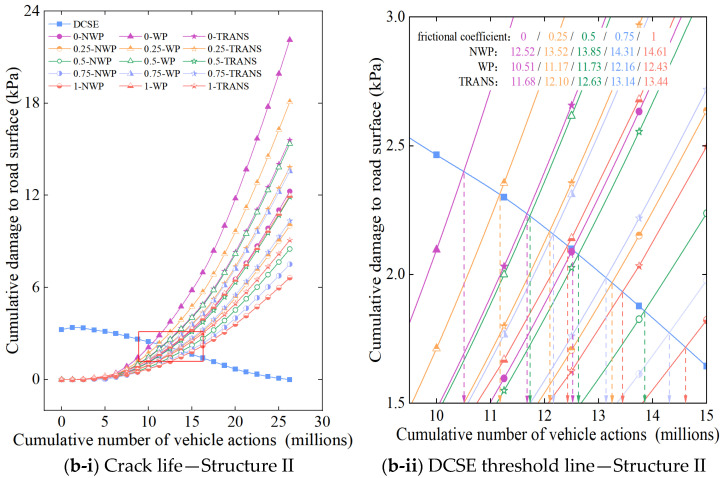
Surfacing (upper) layer crack life is a function of interlayer bonding conditions.

**Figure 16:**
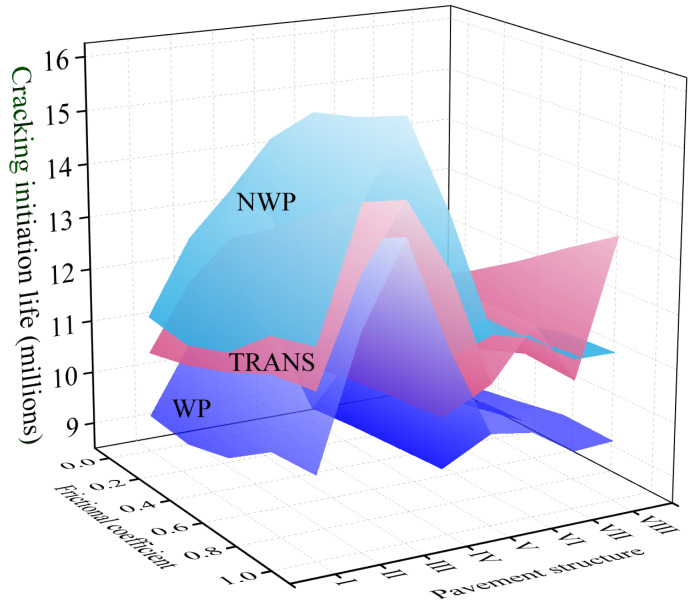
3-D surfacing (upper) layer crack life as a function of interlayer bonding state.

In the 3-D FE model, 0 and 1 were hypothetically assigned to represent the disengaged (i.e., fully unbonded) and fully bonded states between the two successive pavement structural layers. In the study, the friction coefficient was assumed to be between 0 and 1 with a 0.25 incremental sequence to allow for the investigation of the effects of interlayer friction on the pavement stress-strain responses and cracking performance. The lifetimes of crack initiation at each critical location on the top surfacing (upper) layer under different interlayer frictional conditions are shown in Figure 15, where (a) indicates the original pavement Structure I, and (b) indicates the reconstructed pavement Structure II. On the other hand, Figure 16 shows a 3-D plot of the surfacing (upper) layer’s crack life for the WP, NPW, and TRANS locations as a function of the pavement structure and friction coefficient (i.e., interlayer bonding condition).

As depicted in Figure 16, the TDC crack initiation life exhibits a decreasing response trend in the longitudinal NWP zone, the longitudinal WP zone and the TRANS position as the interlayer bonding conditions between the pavement layers gradually deteriorated. Compared with the fully bonded interlayer condition (i.e., friction coefficient = 1.0), the reduction in the crack initiation life in the WP zone is more prominent in pavement Structures II, III, and V, with reduction percentages of 15.45%, 19.10%, and 13.94%, respectively, when fully debonded with a friction coefficient = 0.0. In the NWP zone, the crack initiation life decay is more prominent in pavement Structures III, IV, and V, with the percentage reductions reaching 16.79%, 10.63%, and 10.18%, respectively. For the TRANS location, the decline in the crack initiation life was more prevalent in pavement Structures III, IV, V, VI, and VIII, with the percentage reductions being 14.98%, 11. 86%, 13.33%, 10.62%, and 17.45%, respectively.

Computational results show that under different pavement structures, the crack initiation trend of the TDC in the surfacing (upper) layer is basically consistent with the results of the previous calculations. That is, under different interlayer states, the cracks are still being generated in the WP zone first, and the poorer the interlayer bonding condition, the smaller the crack initiation life is. With the gradual deterioration of the interlayer bonding state (i.e., the friction coefficient regressing towards zero), the degree of decay in the crack initiation life gradually increased and reached a peak reduction when full interlayer failure (i.e., complete debonding) occurred. This is partly due to the tensile stress distribution within the pavement structure being closer when the layers are partially and/or fully bonded. However, when the layers are fully debonded, the respective peaks of both the tensile and shear stresses within the pavement structure increase sharply at localized points—ultimately leading to rapid failure [21]. Therefore, based on the deduced mechanisms of TDC, the crack initiation life in the surfacing (upper) layer of the pavement structure correspondingly decreases when the interlayer bonding state between layers deteriorates.

### 4.5. Energy Ratio (ER) and Crack Analysis

In this study, the ER concept was used to numerically illustrate the effects of fracture damage and quantitatively characterize the evolution of TDC in different model pavement structures. In general, the higher the ER value in magnitude, the better the cracking resistance potential of the asphalt mixture and the corresponding asphalt pavement structure. In the study, the transverse tensile stresses within the pavement layers were calculated using FE analysis under a standard wheel load based on the actual tire-pavement contact pressure (see Figure 2). The DCSEmin calculations, on the other hand, were accomplished by combining the creep compliance parameters of the various respective asphalt mixtures for each corresponding pavement structure and layer, respectively. The results of these ER calculations are summarized in Table 4.

**Figure 17:**
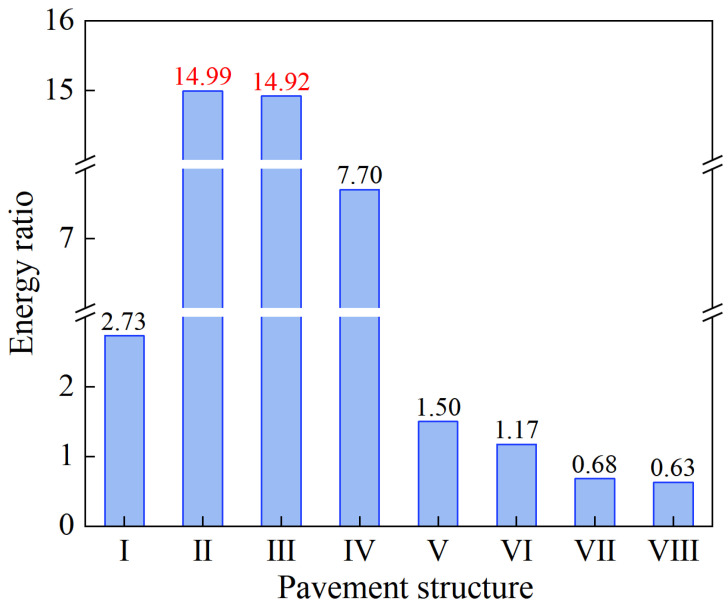
ER, results for the surfacing (upper) layers of different pavement structures.

Table 4, I~VIII in Column One denotes the surfacing (upper) layers of the eight pavement structures under different surfacing layer thicknesses and material combinations. When plotted, the ER response trends are shown in the bar charts in Figure 17.

**Figure 18:**
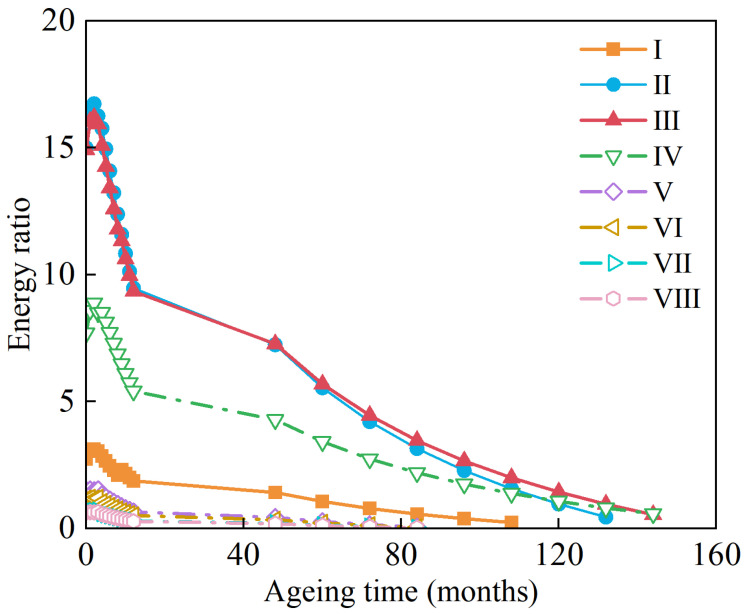
A plot of ER as a function of aging time.

Considering the internal matrix structure and volumetric characteristics of the asphalt mixture itself (i.e., gradation, asphalt binder properties, aggregate properties, etc.) alone, an increase in the energy threshold ($DCSE_{f}$) will inherently lead to an increase in the cracking resistance of the asphalt mixture. Compared to the reconstructed pavement Structures II and III, the energy threshold for the original pavement Structure I at 2.55 kPa was quantitatively the lowest. However, the energy thresholds of the reconstructed pavement Structures II and III are 27.06% and 47.06% higher than those of the original pavement Structure I, respectively. Theoretically, this suggests that the original pavement Structure I has the least ability to resist TDC among the three pavement structures and the top surfacing (upper) layer materials that were evaluated when subjected to similar environmental and loading conditions. The variation in the ER values of the asphalt mixtures at different aging times is illustrated in Figure 18.

As can be seen from the figure, throughout the process of changing with aging time, the ER values of the reconstructed pavement Structures II and III are much larger than that of the original pavement Structure I and the transition pavement Structures IV, V, VI, VII, and VIII, respectively. Comparing pavement Structures II and III from the pre-aging phase through to about 50 months, the ER values of the two pavement structures are comparably overlapping and indifferent. However, with more aging time exceeding 50 months, pavement Structure III tends to exhibit better cracking-resistance performance than pavement Structure II. Furthermore, the ER values of the different pavement structures all show a tendency to increase at zero months and then decrease with aging time. From the previous numerical results in Section 4.2, it was seen that when AC-25 is used for the lower layer (namely pavement Structure I versus IV), the crack initiation life at the critical locations (i.e., 11.87, 14.21, and 13.24 million) is greater than that when ATB-25 is used (i.e., 10.04, 12.30, and 11.52 million), with the ER of pavement Structure IV (2.99) also being greater than that of pavement Structure I (2.55). When SAC-13 is used in the surfacing (upper) layer (namely pavement Structure I versus VI), the cracking life at critical locations (i.e., 10.07, 11.80, and 12.05 million) is comparable to that of SMA-16 (i.e., 10.04, 12.30, and 11.52 million), with the ER of pavement Structure I (2.99) also being greater than that of pavement Structure VI (2.55).

To summarize, considering the asphalt mixture properties and the pavement structural characteristics, the reduction in the surfacing (upper) layer thickness and change in surfacing (upper) layer materials resulted in a 61.35% increase in the ER of the rebuilt pavement Structure II and about 71.17% increase in the ER of the rebuilt pavement Structure III compared to the original pavement Structure I. Without a doubt, this theoretically suggests that the reconstructed pavement Structures II and III have better resistance to cracking than the original pavement Structure I.

## 5. Conclusions

This numerical study conducted modeling and simulative calculations on eight different hypothetical pavement structures, including the original (the municipal main road), two reconstructed, and five transition pavement structures. The study investigated the impact of different pavement structures and materials and various influencing parametric variables on TDC evolution and crack life. From the study results and findings, the main conclusions drawn are as follows:

(1) Macroscopically, cracks always first appeared at the WP, as theoretically expected. Among the eight pavement structures evaluated, the surfacing (upper) layer was consistently the first to develop cracks. Based on field investigations and numerical simulation results, it can be concluded that the type of cracks prevalent on the municipal main roads in Urumqi (China) is mostly TDC.

(2) Under the combination of the original surfacing (upper) layer thickness of 5/7/8 cm, replacing the underlying layer material ATB-25 with AC-25 significantly increases the cracking life—with a recorded increment of 16.23%, 13.77%, and 11.06% in the WP zone, NWP zone, and TRANS location, respectively. Furthermore, when the upper SMA-16 layer was replaced with SBS AC-13, whilst there was a slight reduction in cracking life in the NWP zone, an increase was observed in the WP zone and TRANS location. Notably, the change in the middle layer was more significant, with an average increase of 27.91%. Overall, using the AC-25 asphalt mixture instead of ATB-25 is structurally beneficial for improving resistance to TDC.

(3) Under the combination of the 4/6/7 cm reconstructed pavement structure, replacing SMA-16 with the slightly softer SMA-13 in the surfacing (upper) layer and ATB-25 with AC-25 in the lower layer led to a significant increase in the TDC life over the original pavement structure, with an average increment of 19.75%, 17.76%, and 10.46%, respectively. Compared with SBS AC-13, SMA (due to its superior mix-design volumetrics) was found to be more suitable for use as the surfacing (upper) layer material to mitigate TDC.

(4) After considering the effects of UV aging, the change pattern and response trends of the cumulative loaded-induced pavement damage were basically the same as those without UV aging consideration. In the different pavement structures evaluated, the TDC life in the different pavement layers exhibited a decreasing response trend with the influence of UV aging.

(5) Under different interlayer bonding conditions, cracks still appear in the WP zone first, with the crack initiation life exhibiting a correspondingly decreasing response trend as the interlayer bonding state gradually detdeterioratesn general, the poorer the interlayer bonding condition, the poorer the pavement structural integrity and the shorter the crack life.

(6) Based on its lower ER values, the findings indicated that the original pavement structure was less resistant to cracking when under similar environmental and loading conditions. When subjected to UV aging, the reconstructed pavement structures still exhibited superior performance with higher ER values than the original pavement structure. Whilst an increasing trend was noted in the pre-aging phase, the ER generally exhibited a declining response trend with aging time. For the two modified pavement structures, their ER values were comparably overlapping in the pre-aging phase, with pavement Structure III exhibiting superior performance (i.e., higher ER values) and better cracking resistance with more aging time exceeding 50 months.

This study analytically explored the evolution of TDC in roads in Urumqi (China) using seven different asphalt mixtures and two pavement structural layer thickness combinations. Therefore, more targeted research is still needed to enhance the model parameters and improve the reliability of the TDC prediction model in future follow-up studies. Model verification under different environmental and loading conditions, along with more field validation studies, are also recommended. Notably, the simulated cracking patterns showed qualitative consistency with field observations in Urumqi’s freeze-thaw environments, confirming the model’s applicability to regional conditions. Nonetheless, the study serves as a viable reference datum for numerically predicting the TDC life of asphalt pavements under various loading and environmental conditions. Additionally, the study also provides some insights into the potential materials, anti-aging agents, and pavement structural layer combinations to minimize TDC. Future studies may integrate machine learning with mechanical modeling to enhance practicality. For instance, combining finite element results with historical inspection data could train neural networks for rapid TDC prediction.

## Figures and Tables

**Table 1:** Asphalt-binder logarithmic viscosity ratio at different temperatures.

Temperature		10 °C	30 °C	45 °C	60 °C	75 °C
Logarithmic viscosity ratio $\frac{\mathrm{lg}\eta_{1}}{\mathrm{lg}\eta_{2}}$ (cP)	Base asphalt	0.99	1.05	1.08	1.11	1.08
	Modified asphalt	1.27	1.06	1.06	1.07	1.05

**Table 2:** Pavement structures evaluated.

Structure	Upper Layer	Middle Layer	Lower Layers	Cement Stabilized Aggregate Base	Sub-Base
I-Original pavement structure	5 cm SMA-16	7 cm AC-20	8 cm ATB-25	36 cm	30 cm natural gravel
II-Reconstruction structure A	4 cm SMA-13	6 cm AC-20	7 cm AC-25	39 cm	
III-Reconstruction structure B	4 cm SMA-13 (4% anti-aging agent)	6 cm SBS AC-20	7 cm AC-25 (0.3% anti-peeling agent)	39 cm	
IV-Transition structure ①	5 cm SMA-16	7 cm AC-20	8 cm AC-25	36 cm	
V-Transition structure ②	5 cm SAC-13	7 cm AC-20	8 cm ATB-25	36 cm	
VI-Transition structure ③	5 cm SAC-13	7 cm AC-20	8 cm AC-25	36 cm	
VII-Transition structure ④	4 cm SAC-13	6 cm AC-20	7 cm ATB-25	39 cm	
VIII-Transition structure ⑤	4 cm SAC-13	6 cm AC-20	7 cm AC-25	39 cm	

Legend: SAC-13 = SBS modified AC-13.

**Table 3:** Material input parameters used in the TDC model.

Material Type	Creep Compliance Parameter		Resilience Modulus (MPa)	Dynamic Modulus (MPa)	Fracture Energy (kJ/m^3^)	Effective Asphalt Binder Content (%)	Air Voids (%)
	*m*	*D*_1_					
SMA-13	0.529	7.50 × 10^−5^	2643	7586	5.26	6.1	3.7
AC-20	0.547	1.65 × 10^−5^	3016	8957	5.19	4.4	4.1
AC-25	0.518	3.22 × 10^−5^	4275	13,381	7.12	3.9	4.1
SAC-13	0.531	4.62 × 10^−5^	2818	8739	7.47	5.0	4.2
SMA-16	0.523	6.38 × 10^−5^	2767	7885	4.80	5.6	4.0
ATB-25	0.546	1.91 × 10^−5^	3595	10,731	4.19	3.5	3.8

**Table 4:** ER, parametric results under actual tire-pavement contact pressure.

Pavement Structure	m-Value	D_1_ (10^−4^) (1/kPa)	Stress, σ (kPa)	$DCSE_{\min}$ (kPa)	$DCSE_{f}$ (kPa)	ER
I	0.523	0.64	226	0.934	2.55	2.73
II	0.529	0.75	127	0.216	3.24	14.99
III	0.534	0.75	133	0.252	3.75	14.92
IV	0.523	0.64	172	0.388	2.99	7.70
V	0.531	0.46	210	3.072	4.61	1.50
VI	0.531	0.46	228	3.923	4.61	1.17
VII	0.531	0.46	273	6.769	4.61	0.68
VIII	0.531	0.46	279	7.264	4.61	0.63

## Data Availability

The original contributions presented in this study are included in the article. Further inquiries can be directed to the corresponding author.
